# Reinforced PEI/PVdF Multicore-Shell Structure Composite Membranes by Phase Prediction on a Ternary Solution

**DOI:** 10.3390/polym10040436

**Published:** 2018-04-13

**Authors:** Jihye Chae, Sejoon Park, Dong Young Kim, Han-Ik Joh, Jong Man Kim, Sungho Lee, Seong Mu Jo

**Affiliations:** 1 Center for Materials Architecturing, Korea Institute of Science and Technology,14 Gil 5 Cheongryang-ro, Seongbuk-gu, Seoul 02792, Korea; cjh870725@hanmail.net (J.C.); dykim@kist.re.kr (D.Y.K.); 2 Department of Chemical Engineering, Hanyang University, 222 Wangsimni-ro, Seongdong-gu, Seoul 04763, Korea; jmk@hanyang.ac.kr; 3 Carbon Composite Materials Research Center, Korea Institute of Science and Technology, 92 Chudong-ro, Bongdong-eup, Wanju-gun, Jeollabuk-do 55324, Korea; sejoon.park@kist.re.kr; 4 Department of Energy Engineering, Konkuk University, 120 Neungdong-ro, Gwangjin-gu, Seoul 05029, Korea; hijoh@konkuk.ac.kr; 5 Department of Nano Material Engineering, Korea University of Science and Technology, 217 Gajeong-ro, Yuseong-gu, Daejeon 34113, Korea

**Keywords:** electrospinning, multicore-shell, polymer blend, membrane, Flory–Huggins theory

## Abstract

To construct a polyetherimide (PEI)-reinforced polyvinylidene fluoride (PVdF) composite membrane with multicore-shell structure, a ternary solution was prepared and electrospun by single-nozzle electrospinning. A theoretical prediction was made for the feasibility of complete distinction of two phases. The diameters of the membrane fibers and the PEI multi-core fibrils varied with the PEI ratio and the spinning time, respectively. The tensile strength and modulus were improved to 48 MPa and 1.5 GPa, respectively. The shrinkage of the membrane was only 6.6% at 180 °C, at which temperature the commercial PE separator melted down. The reinforcement in mechanical and thermal properties is associated with multiple PEI nanofibrils oriented along the fiber axis. Indeed, the unique morphology of self-assembled multicore-shell fibers plays an important role in their properties. All in all, PEI/PVdF membranes are appropriate for a lithium-ion battery application due to their high mechanical strength, excellent thermal stability, and controllable textural properties.

## 1. Introduction

Electrospinning is one of the methods used to draw fine (usually a few micrometers to nanometers) fibers using electrostatic force [1]. The electrospinning process consists of a syringe to hold the polymer solution or polymer melt, two electrodes (the needle serves as one of the electrodes), and a high direct current (DC) voltage supply in the kV range and a collector. When sufficient voltage to overcome the viscosity and the surface tension is applied to a polymer drop, the polymer drop from the tip becomes charged and stretched without breaking up due to the polymer entanglements within the solution, the liquid stream from the surface erupts at a critical point, and the point of eruption is the Taylor cone. If the polymer droplet has sufficient viscosity, a charged jet is formed. The charge causes the fibers to bend and then the electrostatic repulsion initiates. It causes a whipping process which elongates the liquid polymer jet. The fibers are finally deposited on the collector. The elongation and the thinning of the fibers caused by the bending instability form uniform fibers with nano or micrometer-scale diameters [2,3]. Especially, nanofiber membranes with a core-shell structure from the electrospinning process have been widely researched due to their high possibilities for various applications, such as scaffolds, drug delivery systems, and sensors [4,5,6,7]. The conventional way to create a core-shell structure in electrospun fibers is to conduct electrospinning with two kinds of polymer solutions though a coannular nozzle and have the core polymer solution and the shell polymer solution flow through the inner tube and the outer tube, respectively. Bazilevsky et al. reported that the production of multicore-shell nanofibers from a single nozzle was successfully accomplished by processing a solution with two distinct phases from immiscible polymer blends [8].

Electrospun nanofiber membranes of PVdF have been studied as one of the good candidates for lithium-ion battery separators because PVdF is easily processable and is a piezoelectric fluoro polymer with good affinity toward electrolytes [9]. Also, it possesses higher porosity, better ionic conductivity, and wider electrochemical stability windows compared to the conventional separators [10,11,12,13,14,15]; however, PVdF still lacks the thermal stability and mechanical strength required in order to be applied in various commercial applications. PEI with ether and imide linkages is one of the high-performance thermoplastics due to its aromatic rings rendering stiffness [16,17,18]. According to Bastida et al., the ether linkages lead to good melt-flow characteristics and processability [17]. Another interesting feature is its excellent thermal stability, because PEI has a high glass transition temperature at ~220 °C [18]. Therefore, composites with PEI as the polymer matrix have been widely studied [19,20,21,22].

In this study, to compensate for such drawbacks of PVdF and integrate a reinforcing effect from the thermal/mechanical stability of PEI by a simple manufacturing process, we prepared a single-nozzle electronspun PEI/PVdF membrane with a ternary blend solution, two polymers and one solvent, using multi-core shell phase separation. Many parameters, such as the blend ratio, the molecular weight, and the chi interaction, influence the architecture of a multicore-shell structure, which would determine the membrane’s stability and performance in application. Therefore, a qualitative estimation on blending was calculated along with the experiment. Specifically, the Flory–Huggins theory was used to understand the polymer blend phase separation for membrane forming or fiber manufacturing [23,24,25]. These applications often require the analysis of a ternary or quaternary system. The theoretical computation by the Flory–Huggins theory brought us to generate a phase diagram describing the phase separation behavior of the solution and the desired architecture of the fiber.

## 2. Theory and Computation

Our purpose of constructing multicore PEI fibrils in PVdF fibers is not only to preserve the electrochemical advantage of PVdF, at the same time, but to reinforce the PVdF membrane by introducing a PEI component as multicore fibrils. For an optimal result, the membrane should take PVdF as a surface component interacting with the electrolyte and PEI as a core component managing the thermal/mechanical stability. Complete distinction between the two components in the solution is required before processing. By conducting the phase prediction on the ternary solution, the composition of the two phases and the fraction of each phase are estimated at three weight ratios. Those determinations would help to inform us about the feasibility of fabricating the multicore-shell structure. The limitations of the classical Flory–Huggins theory are not negligible, and many improvements have been made to the theory taking into account composition fluctuations and monomer architecture. Nevertheless, it has still been successfully used to qualitatively observe the phase behavior of uncharged polymers [26]. In other words, the classical theory is certainly a proper tool to satisfy our aim of computation considering the computational effort required and the appropriate information. The Flory–Huggins theory for the Gibbs energy of mixing for a ternary system (1-PEI, 2-PVdF, and s-DMAc) is expressed as follows [27]:

(1) $$\frac{\Delta G_{m}}{RT}{=n}_{1}\ln\emptyset_{1}{+n}_{2}\ln\emptyset_{2}{+n}_{s}\ln\emptyset_{s}{+(x}_{12}\emptyset_{1}\emptyset_{2}{+x}_{1s}\emptyset_{1}\emptyset_{s}{+x}_{2s}\emptyset_{2}\emptyset_{s})(m_{1}n_{1}+m_{2}n_{2}{+m}_{s}n_{s})$$

where *n*_i_ and Ø_i_ are the number of moles and the volume fraction of the i-th component, respectively; *x*_ij_ is the Flory–Huggins chi interaction parameter between the *i*-th and *j*-th components; and m_i_ is the molar volume ratio of the *i*-th component to the solvent. Therefore, *m*_s_ is equal to 1 because the reference material is itself. The number-average molecular weights of PEI and PVdF were used to estimate the molar volume ratio between three components. The chi parameter, which is a function of temperature and the ratio of two interacting components, was assumed to be a constant in this case for simplicity. The chi parameters were directly estimated from the Hansen solubility parameter, which considers the dispersion, polar, and hydrogen bonding interaction separately (Table 1). By considering the difference of three kinds of interactions, the equation used was:

(2) $$x_{\mathrm{ij}}=VA_{i,j}/RT$$

where $A_{i,j}{=[(\delta }_{\mathrm{Dj}}-\delta_{\mathrm{Di}}{)}^{2}+{0.25(\delta }_{\mathrm{Pj}}-\delta_{\mathrm{Pi}}{)}^{2}+{0.25(\delta }_{\mathrm{Hi}}-\delta_{\mathrm{Hj}}{)}^{2}]$ and *V* is the molar volume [28]. The chemical potential can be derived by differentiating the given Gibbs free energy of mixing (Equation (1)) with respect to each mole of component [27]. Since the system of interest contains three components of two polymers and one solvent, three chemical potential equations are available as follows:

(3) $$\frac{\Delta \mu_{1}}{RT}=\ln\emptyset_{1}{+m}_{1}{[X}_{1}{\left(\emptyset_{2}+\emptyset_{s}\right)}^{2}{+X}_{2}\emptyset_{2}{}^{2}{+X}_{s}\emptyset_{s}{}^{2}]+\left[1-\left(\frac{m_{1}}{m_{2}}\right)\right]\emptyset_{2}+(1-m_{1})\emptyset_{s}$$

(4) $$\frac{\Delta \mu_{2}}{RT}=\ln\emptyset_{2}{+m}_{2}{[X}_{2}{\left(\emptyset_{1}+\emptyset_{s}\right)}^{2}{+X}_{1}\emptyset_{1}{}^{2}{+X}_{s}\emptyset_{s}{}^{2}]+\left[1-\left(\frac{m_{2}}{m_{1}}\right)\right]\emptyset_{1}+(1-m_{2})\emptyset_{s}$$

(5) $$\frac{\Delta \mu_{s}}{RT}=\ln\emptyset_{s}{+X}_{s}{\left(\emptyset_{1}+\emptyset_{2}\right)}^{2}{+X}_{1}\emptyset_{1}{}^{2}{+X}_{2}\emptyset_{2}{}^{2}+(1-\frac{1}{m_{1}})\emptyset_{1}+(1-\frac{1}{m_{2}})\emptyset_{2}$$

where

$$X_{1}=0.5\left(x_{1s}{+x}_{12}-x_{2s}\right)$$

$$X_{2}=0.5\left(x_{2s}{+x}_{12}-x_{1s}\right)\;$$

$$X_{s}=0.5\left(x_{1s}{+x}_{2s}-x_{12}\right)$$

$$m_{1}{=V}_{1}{/V}_{s}$$

$$m_{2}{=V}_{2}{/V}_{s}$$

where *V*_i_ is the molar volume of the *i*-th component.

At the equilibrium, the chemical potential of each component in two different phases should be equal:

(6) $${\Delta \mu }_{i,A}\left(\emptyset_{1,A},\emptyset_{2,A},\emptyset_{s,A}\right){=\Delta \mu }_{i,B}\left(\emptyset_{1,B},\emptyset_{2,B},\emptyset_{s,B}\right),\;i=1,\;2,\;s$$

where A and B represent one phase and the other, respectively.

Also, the sum of the volume fractions in two phases should be equal to 1 by the material balance:

(7) $$\sum \emptyset_{i,A}=\sum \emptyset_{i,B}=1,\;i=1,\;2,\;s$$

Equations (6) and (7) are a set of five coupled nonlinear equations. The two material balance equations were combined with Equation (6) to eliminate the two unknowns and leave three equations. One unknown of one phase needs to be specified to solve the other five unknowns. To avoid trivial solutions where the volume fraction of each component is equal, a penalty function to prompt a large residual near the trivial solution was defined, and the difference of potential function was divided by the difference of fractions to either a power of *p* = 2 or 4 depending on how far it was from the immiscible limit point. The objective function to be minimized was defined as:

(8) $$\mathrm{OBJ}=\sum \frac{{{(\Delta \mu }_{i,A}-{\Delta \mu }_{i,B})}^{2}}{{\left(\emptyset_{i,A}-\emptyset_{i,B}\right)}^{p}{\left(RT\right)}^{2}}.$$

For each computation trial, the choice of initial guess on equilibrium compositions was made for efficient computation towards a solution by setting the initial guess within a reasonable range of compositions. The criteria of convergence to the solution was set as OBJ < 10^−7^. The PEI-rich phase volume fraction with respect to the polymer blending ratio determines the diameter of reinforcing columns, PEI fibrils, inside the membrane fiber. To qualitatively estimate the growth of PEI-rich phase with respect to the blending ratio of two polymers, total volume fractions and the equilibrated phase volume fractions of each component were related as follows:

(9) $$P_{\mathrm{PEI}}=\frac{x_{i}-z_{i}}{x_{i}-y_{i}}$$

where *P*_PEI_ is the volume fraction of PEI-rich phase in the total mixture; *x*_i_ is the equilibrated volume faction of *i* component in PVdF-rich phase; *y*_i_ is the equilibrated volume fraction of *i* component in PEI-rich phase; and *z*_i_ is the volume fraction of *i* component in the working mixture.

## 3. Experimental Details

### 3.1. Spinning Solution

Commercial PEI (ULTEM^TM^ 1000, *M*_n_ = 21 kDa, *M*_w_ = 54 kDa, Sabic, Riyadh, Saudi Arabia) [29] and PVdF (Kynar^®^ 761, *M*_n_ = 145 kDa [30], *M*_w_ = 441 kDa [31]) were firstly placed in a vacuum drying oven at 60 °C for 24 h. All working solutions were prepared with computationally verified compositions for phase separation. To investigate the physical properties of the membrane as a function of the polymer ratio, polymers with three different weight ratios of 2:1, 1:1, and 1:2 (=PVdF:PEI) were dissolved in the dimethylacetamide (DMAc) solvent.

### 3.2. Membrane Preparation

The electrospinning was conducted at room temperature. The fluid was fed at 30 mL/min into the syringe (30 G, I.D. = 0.15 mm, O.D. = 0.30 mm) by an infusion/withdrawal pump. The syringe needle was subjected to an electrical potential of 11.5 kV. The tip-to-ground distance was 15 cm. The whipping fibrous matrix from the Taylor cone was deposited on a stainless steel collector. During the electrospinning process, the collector was replaced with a new one every 10 min. The total time of deposition was 40 min, which implies that four membranes were prepared in one working solution. The four membranes were recorded as PEI/PVdF_10min, PEI/PVdF_20min, PEI/PVdF_30min, and PEI/PVdF_40min and were named after their collection times. The electrospun membranes were placed in the vacuum drying oven at 60 °C for 12 h to remove leftover solvent. The average thickness of the membranes was about 80 μm. Post processes, such as hot-pressing at 160 °C and heat-stretching at 225 °C, were conducted. As a result, the size of the thickness was reduced to 20 μm. The membranes were dried in a vacuum oven at 60 °C to evaporate remaining solvent, then placed in a glove box filled with Ar gas (H_2_O < 1 ppm) prior to measurement. To observe the PEI structure in a fabricated fiber, membrane samples were prepared by 0.5 × 0.5 cm^2^. The samples were placed in a glass bottle of 50 mL acetone. The samples were maintained at 60 °C overnight and vacuum-dried for 24 h to remove PVdF.

### 3.3. Characterization

To physically confirm the existence of multiple PEI emulsions with the working composition set by the theory, the morphology of the blend solution was observed via an optical microscope (OM, Zeiss polarizing, built-in automatic camera, Zeiss, Oberkochen, Germany). One drop of sample solution was placed on a slide glass and observed with a function of time. The PVdF extraction of the membrane was conducted to selectively examine the multi-core structure of the PEI by placing the 0.5 × 0.5 cm^2^ of the as-spun membrane in a glass bottle of 50 mL acetone at 60 °C overnight. The morphologies of both the as-electrospun membrane and the PVdF-extracted membrane were observed via Field emission scanning electron microscopy (FE-SEM, Hitachi, Tokyo, Japan, S-4200) under vacuum conditions. All membrane samples were Pt/Pb-coated for 2 min before loading. Based on OM and SEM images, average diameters of both emulsions and nanofibers in the membrane were calculated via the Image-Pro Plus 4.5 software (Media Cybernetics Inc., Rockville, MD, USA). A universal testing instrument (Instron Model 4464, Instron, Norwood, MA, USA) was used to measure the mechanical properties of the PEI/PVdF blend membrane and PE separator at room temperature. The cross-head speed was 10 mm/min and the dimension of the samples was 6.0 × 0.5 cm^2^. The test was conducted based on the ASTM standard D882-95a. The thickness of the samples was measured with a dial gauge (Mitsutoyo, Kawasaki, Japan, BWB836) in advance of tensile measurement. To examine the thermal stability of the PEI/PVdF blend membrane and PE separator, thermo-mechanical analysis (TMA, Perkin-Elmer, Waltham, MA, USA, TMA-7) was conducted to investigate the thickness change as a function of temperature. Samples were cut into dimensions of 2 × 0.5 cm^2^ and heated from 50 to 250 °C at the rate of 10 °C/min with a static force of 50 mN. To observe thermal shrinkage, the PEI/PVdF membrane and PE separator were cut into dimensions of 3 × 3 cm^2^ and stored in a furnace for 1 h with temperatures of 180, 200, 220, and 240 °C. The dimensional change was measured after the samples were taken from the furnace.

The apparent porosity was calculated using the following equation:

(10) $$P(\%)=\left(1-\frac{\rho_{m}}{\rho_{p}}\right)\times 100$$

where P, ρ_m_, and ρ_p_ are the apparent porosity, density of the membrane, and polymer density, respectively. The electrolyte uptake of the electrospun membrane was determined using Equation (10). The membrane was impregnated into the electrolyte solution for 1 h and then the remaining electrolyte on the surface was removed by wiping it with filter paper. The mass of the electrolyte absorbed into the membrane after wiping was measured. The weights of the membrane before and after impregnation into the electrolyte solution were measured. The equation follows:

(11) $$\mathrm{uptake}(\%)=\left(\frac{M-M_{0}}{M_{0}}\right)\times 100$$

where *M* and *M*_0_ are the mass of the membrane-absorbing electrolyte and the mass of the membrane, respectively.

The pore size and distribution of PEI/PVdF blend membranes were measured using a Capillary Flow Porometer (Porous Materials Inc., CFP-1500 AEL, Newtown Square, PA, USA). In a dry-up process, some air penetrated the membrane. Subsequently, Galwick with 15.9 dynes/cm as an absorbing solvent was spread over the membrane in a wetting process and the air was passed again. The pore size and distribution were detected and calculated by comparing the gas flow rates between the drying and wetting processes at the same pressure. Gurley number, also measured by PMI, indicates the air permeability of separators and is related to the porous structure of membranes.

## 4. Results and Discussion

### 4.1. Phase Separation in Ternary Solution

**Figure 1:**
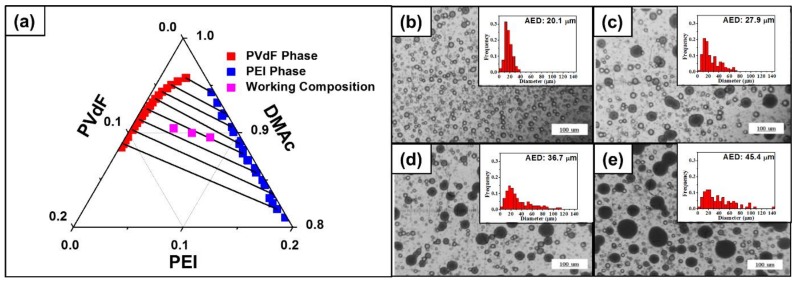
(**a**) Computed bimodal curve and tie lines of two polymers, PEI and PVdF, in DMAc solvent and optical microscope (OM) images and PEI droplet size distribution of PEI/PVdF (1:1 wt. ratio) blend solution after a waiting time of (**b**) 10 min; (**c**) 20 min; (**d**) 30 min; and (**e**) 40 min without stirring. The frequencies in insets were normalized by the number of counted samples. AED is the abbreviation for average emulsion diameter.

Figure 1a represents the binodal curve and the tie lines of the ternary phase diagram expressed in volume fraction. Anticipating features of the ternary solution can be pointed out with the diagram. Working compositions of all weight ratios (PVdF:PEI = 2:1, 1:1, and 1:2) lie inside of the binodal curve, which implies that the working solution would have two separate phases. Considering the degree of the difference between the chi parameters of the three pairs, each component definitely favors or disfavors a certain component with the result that the immiscible region occupies most of the phase diagram (immiscible limit ~0.96). In addition, the diagram indicates that there would be a complete distinction between PEI- and PVdF-rich phases. The curve is slightly shifted toward the PEI direction, leaving space of miscible region upon the PVdF-rich phase curve. This region originated from the unequal molar volume between the polymers (*m*_PEI_ < *m*_PVdF_). Furthermore, the tie lines are tilted toward the PVdF-rich corner, representing that the solvent (DMAc) prefers staying in PVdF-rich phase rather than in PEI-rich phase (*X*_DMAc,PVdF_ << *X*_DMAc,PEI_). This solvent distribution of DMAc to the PVdF allows for building a more dense PEI structure, which would serve as a reinforcing column. The immiscibility of the mixture of the three weight ratios was also verified with the physical observation by OM. OM images of a mixture droplet (PVdF:PEI = 1:1) taken at 10 min intervals for 40 min are shown in Figure 1b–f. For all time intervals, the two distinct phases are observed. The size of emulsions was gradually increased with more waiting time, indicating that the phase separation and the coalescence of PEI emulsions were being preceded. An increment in size of the PEI emulsions was also observed with respect to not only the time, but also the PEI weight ratio after the mixing process. The OM images of the mixture droplet (PVdF:PEI = 1:2 and 2:1) are shown in Figures S1 and S2, respectively, in the Supplementary Information (SI). From the result of the theory using the tie line and the binodal curve, the volume fraction of PEI-rich phase at equilibrium was calculated by Equation (9) for polymer ratios of 1:2, 1:1, and 2:1 (PEI:PVdF); the volume fractions came out to be 0.34, 0.55, and 0.73, respectively. Consequently, as the total mixture composition has richer PEI weight, PEI-rich phase will take a larger portion.

### 4.2. Diameter of Electrospun Fiber

**Figure 2:**
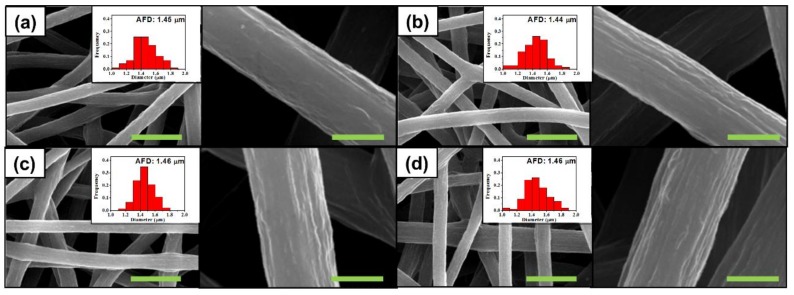
SEM images and diameter distributions of electrospun PEI/PVdF (1:1 wt. ratio) fibers collected after the interval of (**a**) 10 min; (**b**) 20 min; (**c**) 30 min; and (**d**) 40 min. Right images are enlarged for morphology observation. The green scale bars are 6 μm and 1 μm, accordingly. The frequencies in insets were normalized by the number of counted samples. AFD is the abbreviation for average fiber diameter.

As anticipated through theoretical prediction, we designed a multicore PEI-reinforced PVdF membrane using phase separation. All blended solutions were immediately electrospun after the mixing process. Electrospun membranes were taken at 10 min intervals for 40 min from the collector, resulting in four samples. The thickness of membranes was about 80 μm. Figure 2a–d shows SEM micrographs of as-electrospun membranes obtained from 1:1 (=PEI:PVdF) weight ratio solutions after electrospinning for every interval of 10 min, where the insets are the corresponding diameter distributions. All four membranes had diameters of about 1.45 μm with a similar distribution, indicating no significant change in diameter during electrospinning. In contrast, a PEI weight ratio in the blend solutions resulted in significant changes in the diameters of electrospun membranes. The diameters of electrospun fibers were found to be about 1.08 and 1.55 μm with the 1:2 and 2:1 (=PEI:PVdF) weight ratio solutions, respectively (Figures S3 and S4 in SI). However, it was clear that the spinning time did not affect the diameter of fibers, indicating that the diameter of a fiber depends only on the blending ratio and not the spinning time. In other words, a gradual change in the structural organization of PEI emulsions in the solution did not induce a change in a fiber’s diameter.

### 4.3. Morphology of the Fiber

Prior to the experiment, it was anticipated that the PVdF-rich phase would have a greater content of solvent. During the spinning process, shrinkage and coagulation occur. At the same time, there are two distinct phases which have significantly different shrinkage and coagulation. Figure 1a informed us that PVdF-rich phase always contains more solvent; such a characteristic implies that PVdF-rich phase would have more severe surface shrinkage during spinning. Furthermore, as shown in Figure 1a–d, scattered PEI emulsions became agglomerated, leaving more continuous PVdF-rich phase to be shrunken during the spinning. According to Figure 2a–d, the surface roughness became clearer with an increase of time because the solvent portion in the two phases became distinct and the continuous space of PVdF-rich phase was expanded. Similar morphologies were observed with fibers from other blend solutions for the same reason (insets of Figures S3 and S4 in SI).

### 4.4. PEI as a Multiple-Core Fibril

**Figure 3:**
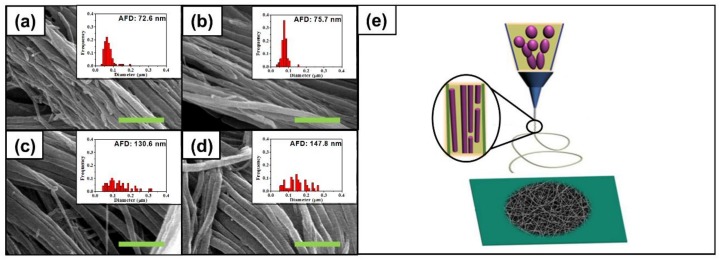
SEM images and diameter distributions of electrospun PEI/PVdF (1:1 wt. ratio) fibers collected after the interval of (**a**) 10 min; (**b**) 20 min; (**c**) 30 min; and (**d**) 40 min and (**e**) schematic showing the formation of membranes containing multicore-shell structured fibers during electrospinning. The green scale bars are 1 μm. The frequencies in insets were normalized by the number of counted samples. AFD is the abbreviation for average fiber diameter.

Figure 3a–d shows PEI fibrils and their diameter distributions after removing PVdF by extraction using acetone with PEI/PVdF (=1:1) blend fibers, indicating that the PEI and PVdF phases were the core and shell components, respectively, in the multicore-shell nanostructured fibers. Since PEI is insoluble in acetone, PVdF components in the electrospun PEI/PVdF blend fibers were dissolved during the extraction and only PEI components remained. The scheme for the multicore-shell structure is shown in Figure 3e. The immiscible mixing solution of PEI and PVdF rendered PEI micro-phase domains in a PVdF matrix in a barrel. During electrospinning, PEI emulsions were highly stretched from the jet. PEI nanofibrils were produced inside PVdF with rapid evaporation of the solvent. In Figure 3a–d, PEI fibrils produced at an initial 10 min had an average diameter of about 73 nm, and the values sequentially increased up to 148 nm. This indicates that mixture taken with a longer time provided thicker PEI fibrils. However, the diameter distribution became broad with samples that were prepared last. Such a gradual change that occurred in the distribution was also observed in the distribution of emulsion diameter as shown in Figure 1a–d. Correspondingly, the size of PEI emulsions influenced the diameters of PEI fibrils even though the diameters of the electrospun PEI/PVdF blend fibers did not change. As expected, more (or less) contents of PEI in blend fibers led to bigger (or smaller) diameters of fibrils as shown in Figures S5 and S6 in the SI. An additional structure examination of electrospun PEI/PVdF_10min was conducted by transmission electron microscopy (TEM) along with energy dispersive x-ray spectroscopy (EDX) on two characteristic elements, nitrogen for PEI and fluorine for PVdF (Figure S7a). Both of the element detection results from EDX scanning showed fluctuation as the scanning position moved over the fiber (Figure S7c,d). Such non-uniform distribution of two elements demonstrates the inhomogeneous composition of the multicore-shell structure.

### 4.5. Mechanical Properties

To prepare more dense membranes, four electrospun membranes from PEI/PVdF (=1:1) blended solutions were pressed at 160 °C, and the thickness of membranes decreased from 80 to 20 μm. The thermally pressed membrane maintained fiber shapes or pores by melting as shown in Figure S8 in the SI. After heat-pressing, the resulting membranes were stretched at 225 °C to increase the orientation of fibers. The membranes also showed pore structure retention upon thermal stretching as presented in Figure S9 in the SI. They showed the maximum stretching ratios of 60%, 89%, 93%, and 100%; this implies that the membrane at the last 10 min interval was stretched more than the other three membranes. The membrane obtained last had a broad distribution of PEI fibrils with a high average diameter after removing PVdF (Figure 3a–d). As the spinning time passed, PEI and PVdF were more separated in the barrel, resulting in the broad diameter distribution with bigger PEI emulsions. Considering that the melting temperature of PVdF is ~165 °C (Table S1) and the glass transition temperature of PEI is ~217 °C (Table S1), it is obvious that heat-stretching at 225 °C mainly depends on PVdF stretching. It is likely that evenly distributed rigid PEI fibrils hindered PVdF stretching, leading to the lowest stretching ratio for PEI/PVdF_10min membranes.

**Figure 4:**
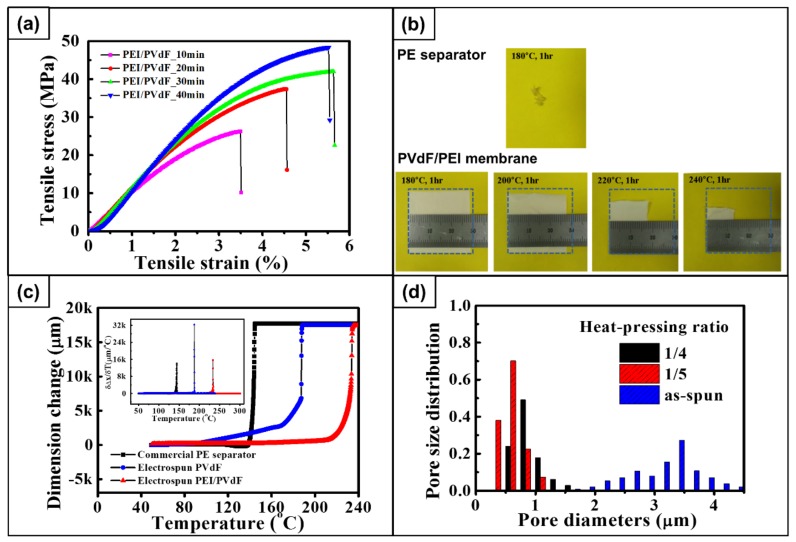
(**a**) Stress-strain curves of the post-treated PEI/PVdF membranes; (**b**) Photographs of commercial PE separator heat-treated at 180 °C and post-treated PEI/PVdF_40min membranes heat-treated at 180, 200, 220, and 240 °C; (**c**) thermo-mechanical analysis (TMA) thermograms of post-treated PEI/PVdF_40min membrane, electrospun PVdF membrane, and commercial PE separator, where the inset is the differentiated dimension; (**d**) Pore size distributions of as-electrospun and post-treated PEI/PVdF_40min membranes.

Tensile properties of heat-stretched membranes in the stretching direction were measured using a universal testing machine. Figure 4a shows the stress-strain curves of each sample measured with a cross-head speed of 10 mm/min. Tensile properties are summarized in Table 2. The tensile strength and modulus of 60% stretched samples (PEI/PVdF_10min) were found to be 26 and 1039 MPa, respectively. The values increased with increasing stretching ratio, and 100% stretched membranes (PEI/PVdF_40min) showed a tensile strength and modulus of 48 and 1475 MPa, which is an 85% and a 42% increase, respectively, compared to 60% stretched membranes. As expected, these values result from more oriented molecules of polymers along the fiber axis. In addition, rigid PEI fibrils in the fibers contribute to the high modulus. This suggests that the morphology of self-assembled multicore-shell fibers plays an important role in mechanical properties.

### 4.6. Thermal Stability

To investigate the dimensional stability of the membranes at high temperature, a thermal shrinkage test was carried out. Figure 4b illustrates the dimensional change of a 100% stretched PEI/PVdF_40min membrane and a commercial PE separator (SKC, Seongnam, Republic of Korea) after heat-treatment in a hot oven at 180 to 240 °C for 1 h. The PE separator completely melted down at 180 °C, whereas the dimensions of the blended membranes slightly shrank by 6.6%. Heat treatment at a higher temperature revealed further shrinkage of the membranes, and significant shrinkage was observed at 220 °C. Finally, only 21% of sample remained at 240 °C. Even though the melting temperature of PVdF is supposed to be 165 °C, a considerable number of membranes did not melt down at 180 °C due to the presence of PEI, which has the glass transition temperature of 217 °C. The high temperature melt integrity (HTMI) was measured by TMA to compare the HTMI property of the PE membrane, the PVdF membrane, and the PEI/PVdF40 membrane as shown in Figure 4c. Dimensional change of the PVdF membrane started at 100 °C and a significant deformation was observed from 165 °C, which is the melting temperature of PVdF. The PE separator began shrinking at 140 °C. At the same time, the PEI/PVdF_40min membrane sustained shrinkage until the temperature had reached 220 °C. From these results, it is clear that PEI/PVdF membranes are thermally stable compared to the PE commercial separator.

### 4.7. Textural Properties

Textural properties of the membranes, such as apparent porosity, flow pore size, average Gurley number, and electrolyte uptake, are listed in Table 3. The apparent porosity of as-electrospun and post-treated PEI/PVdF_40min membranes were 89% and 58%, respectively. Indeed, the pressing treatment reduced the porosity of the membranes by decreasing the pore size. Moreover, a significant change of pore size distribution can be observed in Figure 4d. A Capillary Flow Porometer (Porous Materials, Inc., CFP-1500 AEL, Newtown Square, PA, USA) was used to quantify the permeability of the membranes. The as-electrospun membranes had a mean flow pore size and average Gurley number of 3.26 μm and 0.79 s/100 cc, respectively. Simultaneously, the post-treated membrane revealed that the mean flow pore sizes and average Gurley numbers were 0.81 μm and 6.29 s/100 cc, respectively. The Gurley number indicates the permeability of the separator typically characterized by air. If the porosity becomes higher, the Gurley number is found to be lower. According to Huang and Arora, commercial membranes require a diameter of less than 1 μm and a Gurley number of less than 35 s/100 cc [32,33]. Therefore, the textural properties of the membrane are appropriate for separators. Even though the electrolyte uptake decreased with increasing pressing ratio, the value of 365% should make the membrane applicable as a polymer membrane in lithium-ion batteries.

## 5. Conclusions

PEI and PVdF blended membranes with a multicore-shell structure were prepared by single-nozzle electrospinning of a ternary blended solution. To analyze essential phase separation phenomena of the solution for preparing the multicore structure, a theoretical computation was conducted by the Flory–Huggins theory. The computed diagram was utilized to predict complete separation and solvent distribution between PEI- and PVdF-rich phases. Based on the theoretical prediction, a structure of the PVdF fiber with multiple PEI-reinforcing strings was derived with valid blend ratios. The diameter of the blended fiber in membranes increased from 1.1 to 1.6 µm as the PEI weight ratio increased. The PEI core fibril diameters were measured using PVdF extraction. The PEI core diameters ranged from 65 to 160 nm, respectively, with the increase of the PEI weight ratio and the spinning time. Further investigations on mechanical and thermal properties of the following PVdF/PEI membranes were produced after such post-treatments as heat-pressing at 160 °C and stretching at 225 °C. The most stretched membrane, PEI/PVdF_40min, had the maximum modulus and tensile strength of about 48 MPa and 1.5 GPa, respectively. The post-treated membranes shrunk by only 6.6% at 180 °C, where the commercial PE separator for lithium-ion battery melted down. In addition, TMA thermograms revealed that significant deformation started at 140 and 220 °C with the PE and blend membranes, respectively. The reinforcement in these significant mechanical and thermal properties is associated with oriented molecules of polymers along the fiber axis and thermoplastic PEI fibrils in the fibers during post-processing. In other words, the morphology of self-assembled multicore-shell fibers impacts critically on improving their properties. The membranes with a porosity of 58% and a Gurley number of 6.3 s/100 cc showed 365% electrolyte uptake. Therefore, the PEI/PVdF membranes are appropriate for a lithium-ion battery application due to their high mechanical strength, excellent thermal stability, and controllable textural properties.

## Figures and Tables

**Table 1:** Hansen solubility of DMAc, PVdF, and PEI [28].

Component	Dispersion (MPa^1/2^)	Polar (MPa^1/2^)	Hydrogen Bonding (MPa^1/2^)
DMAc	16.8	11.5	10.2
PVdF	17.0	12.1	10.2
PEI	19.6	7.6	9.0

**Table 2:** Tensile properties of the post-treated PEI/PVdF membranes.

Samples	Tensile Strength (MPa)	Tensile Modulus (MPa)	Strain-to-Failure (%)
PEI/PVdF_10min	26.3	1039	3.5
PEI/PVdF_20min	37.4	1261	4.5
PEI/PVdF_30min	42.0	1328	5.7
PEI/PVdF_40min	48.4	1475	5.5

**Table 3:** Textural properties of as-electrospun and post-treated PEI/PVdF_40min membranes.

Samples	App. Porosity (%)	Mean Flow Pore Size (μm)	Average Gurley Number (s/100 cc)	Uptake with Electrolyte (%)
As-electrospun	89.1	3.26	0.79	475
Post-treated	58.3	0.81	6.29	365

## Supplementary Materials

The following are available online at http://www.mdpi.com/2073-4360/10/4/436/s1.
