# Supplementary material for: Reinforced PEI/PVdF Multicore-Shell Structure Composite Membranes by Phase Prediction on a Ternary Solution
Source: Polymers (Basel). 2018 Apr 13;10(4):436. doi: 10.3390/polym10040436 (PMC6415197; doi:10.3390/polym10040436)
Supplement: Supplementary file 1 [file polymers-10-00436-s001.pdf]

SUPPLEMENTARY INFORMATION

Reinforced PEI/PVdF multicore-shell structure composite membranes by phase prediction on a ternary solution

Jihye Chae,[2‡], Sejoon, Park[3‡], Dong Young Kim[1], Han-Ik Joh[4], Jong Man Kim[2], Sungho Lee[3,5]*, and Seong Mu Jo[1,3]*

[1] Center for Materials Architecturing, Korea Institute of Science and Technology,

14 Gil 5 Cheongryang-ro, Seongbuk-gu, Seoul 02792 Republic of Korea

[2] Department of Chemical Engineering, Hanyang University,

222 Wangsimni-ro, Seongdong-gu, Seoul 04763 Republic of Korea

[3] Carbon Composite Materials Research Center, Korea Institute of Science and Technology,

92 Chudong-ro, Bongdong-eup, Wanju-gun, Jeollabuk-do 55324 Republic of Korea

[4] Department of Energy Engineering, Konkuk University,

120 Neungdong-ro, Gwangjin-gu, Seoul 05029 Republic of Korea

[5] Department of Nano Material Engineering, Korea University of Science and Technology,

217 Gajeong-ro, Yuseong-gu, Daejeon 34113 Republic of Korea

[‡] These authors contributed equally to this paper.

Corresponding Authors: *sunghol@kist.re.kr, *smjo@kist.re.kr

Keywords: Electrospinning, multicore-shell, polymer blend, membrane, Flory-Huggins theory

Table S1. Properties of PEI and PVdF obtained from manufacturer

Properties	PEI	PVdF
Tensile strength (MPa)	85	-
Tensile modulus (GPa)	3.6	-
Vicat softening point (°C)	219	-
Glass transition temperature (°C)	217	-35
Melting temperature (°C)	350	165
Density (g/cm^3)	1.27	1.77

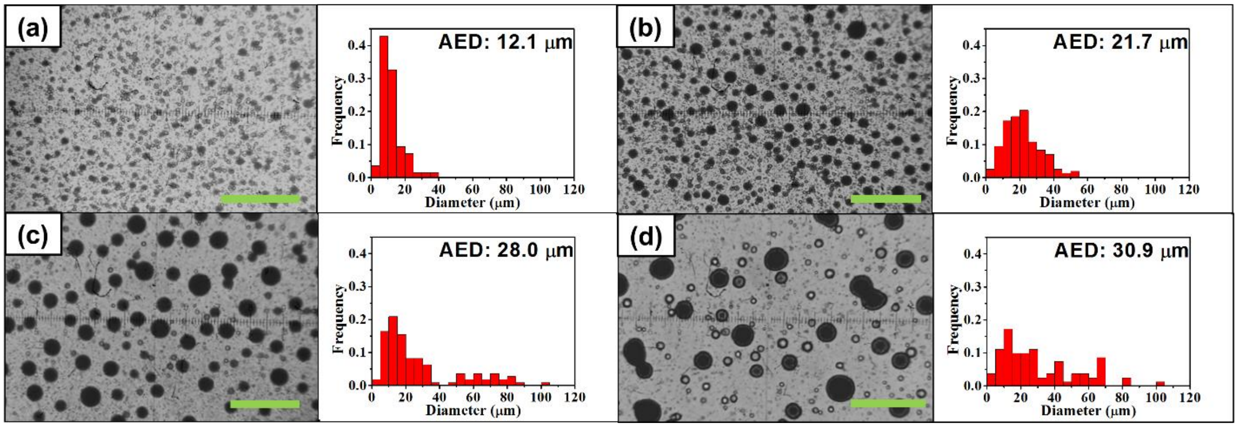

Figure S1. Optical microscope (OM) images and PEI droplet size distribution of PEI/PVdF (1:2 wt. ratio) blend solution after a waiting time of (a) 10 min, (b) 20 min, (c) 30 min, and (d) 40 min without stirring. The green scale bars are 100 μm.

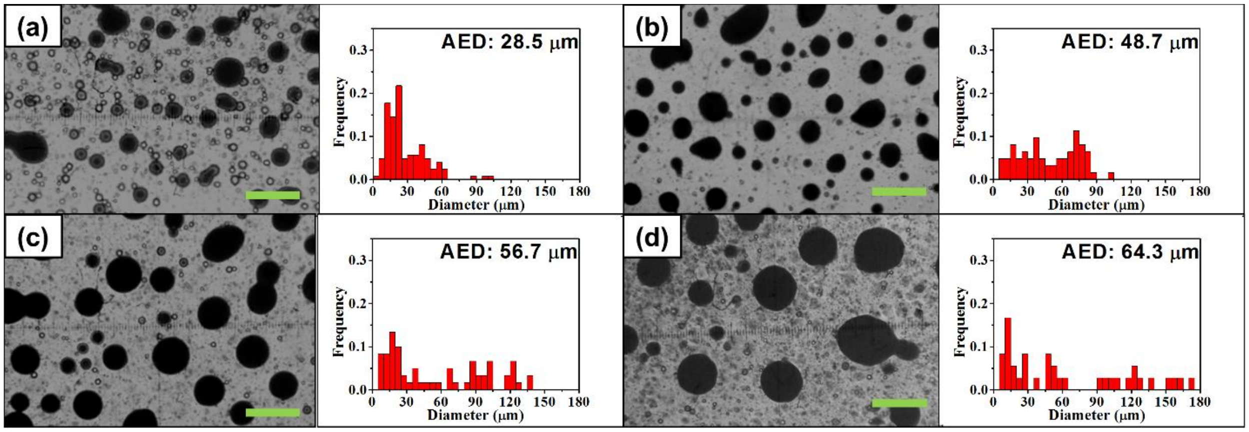

Figure S2. OM images and PEI droplet size distribution of PEI/PVdF (2:1 wt. ratio) blend solution after a waiting time of (a) 10 min, (b) 20 min, (c) 30 min, (d) 40 min without stirring. The green scale bars are 100 μm.

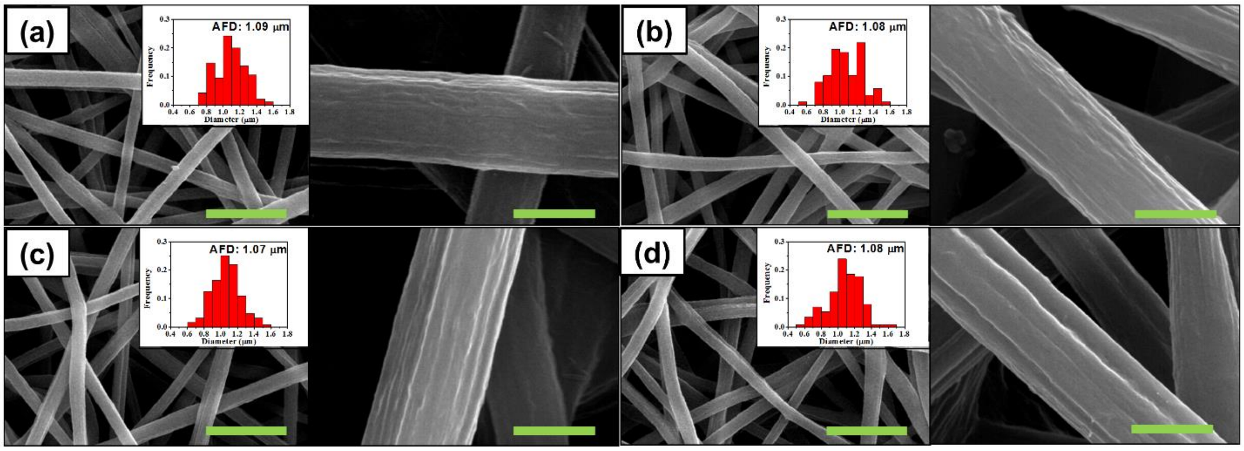

Figure S3. SEM images and diameter distributions of electrospun PEI/PVdF (1:2 wt. ratio) fibers collected after the interval of (a) 10 min, (b) 20min, (c) 30 min, and (d) 40 min. Images on the right are enlarged for morphology observation. The green scale bars are 6 μm and 1 μm accordingly. The frequencies in insets were normalized by the number of counted samples.

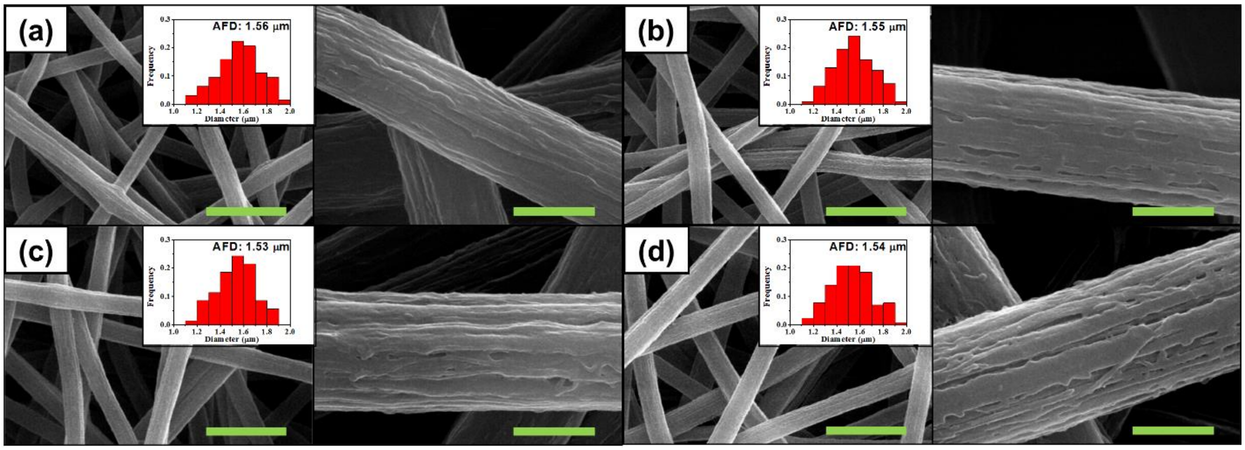

Figure S4. SEM images and diameter distributions of electrospun PEI/PVdF (2:1 wt. ratio) fibers collected after the interval of (a) 10 min, (b) 20min, (c) 30 min, and (d) 40 min. Images on the right are enlarged for morphology observation. The green scale bars are 6 μm and 1 μm accordingly. The frequencies in insets were normalized by the number of counted samples.

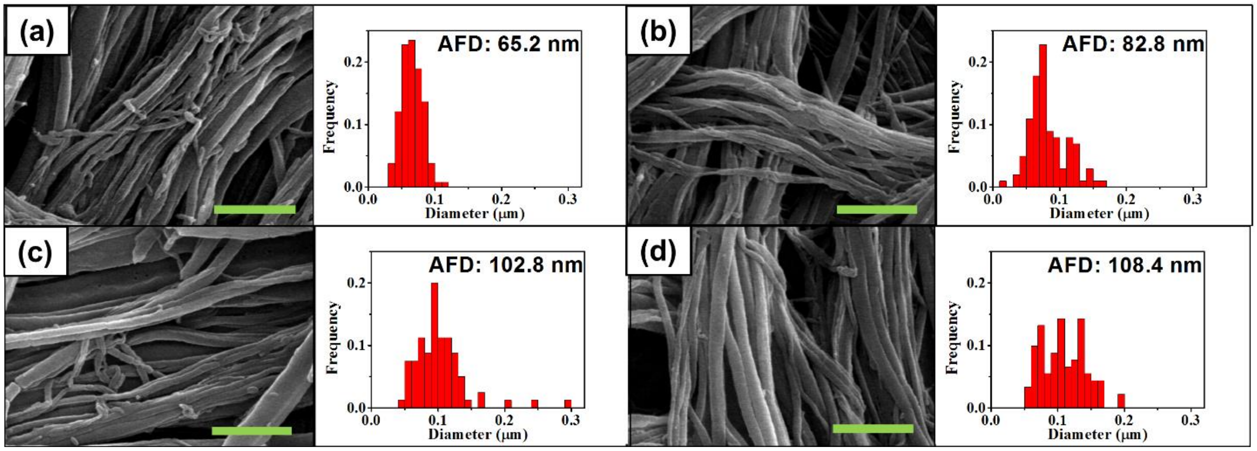

Figure S5. SEM images and diameter distributions of PEI fibrils after PVdF extraction using acetone from electrospun PEI/PVdF (1:2 wt. ratio) fibers collected after the interval of (a) 10 min, (b) 20min, (c) 30 min, and (d) 40 min. The green scale bars are 1 μm.

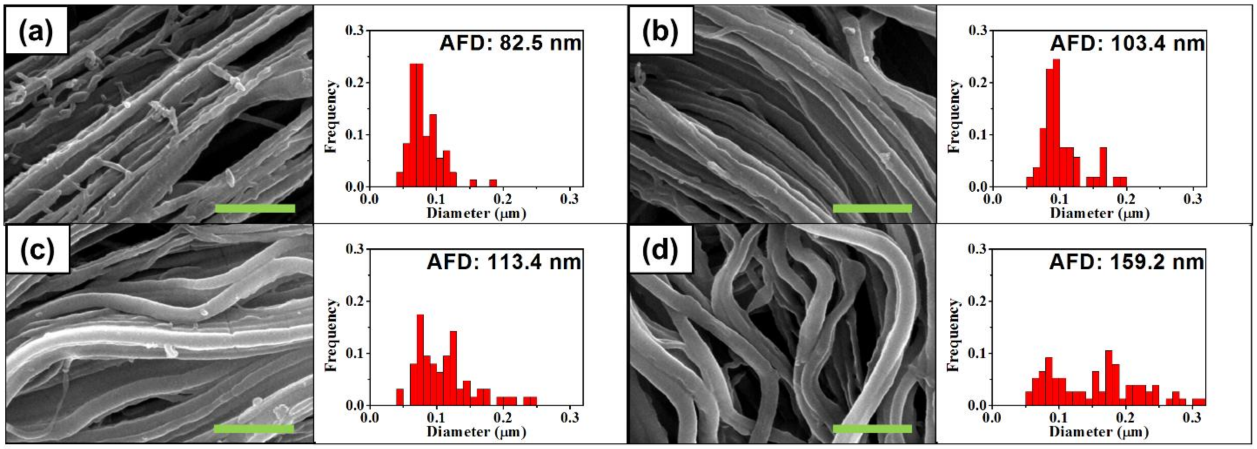

Figure S6. SEM images and diameter distributions of PEI fibrils after PVdF extraction using acetone from electrospun PEI/PVdF (2:1 wt. ratio) fibers collected after the interval of (a) 10 min, (b) 20min, (c) 30 min, and (d) 40 min. The green scale bars are 1 μm.

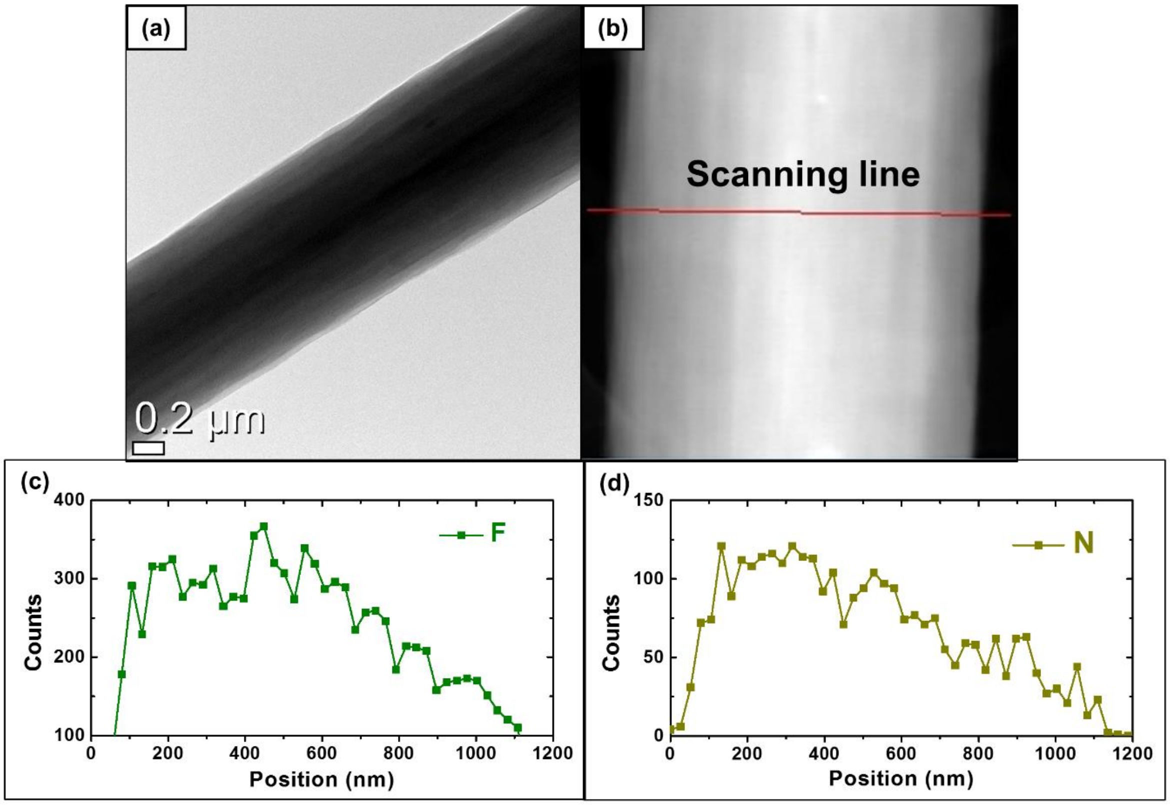

Figure S7. TEM images of (a) PEI/PVdF_10min (1:1 wt. ratio) and (b) EDX scanning line. (c, d) Element detection of fluorine and nitrogen along the scanning line. Fluorine and nitrogen were selected for observing the distribution of PVdF and PEI, respectively.

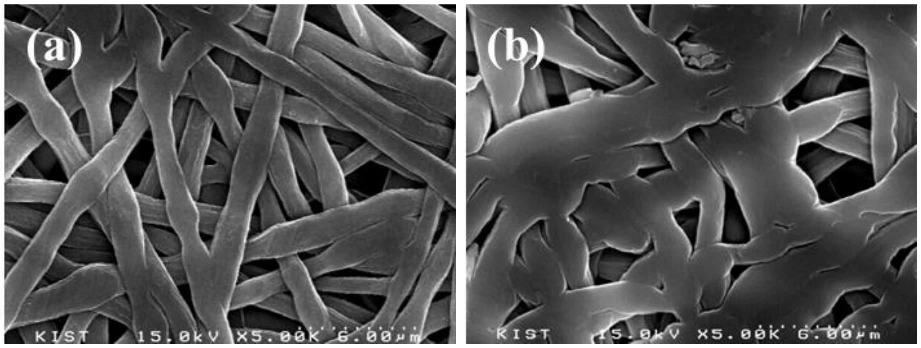

Figure S8. SEM images of the electrospun PEI/PVdF (1:1 wt. ratio) blend membranes after heat-pressing with (a) 1/4 and (b) 1/5 ratios

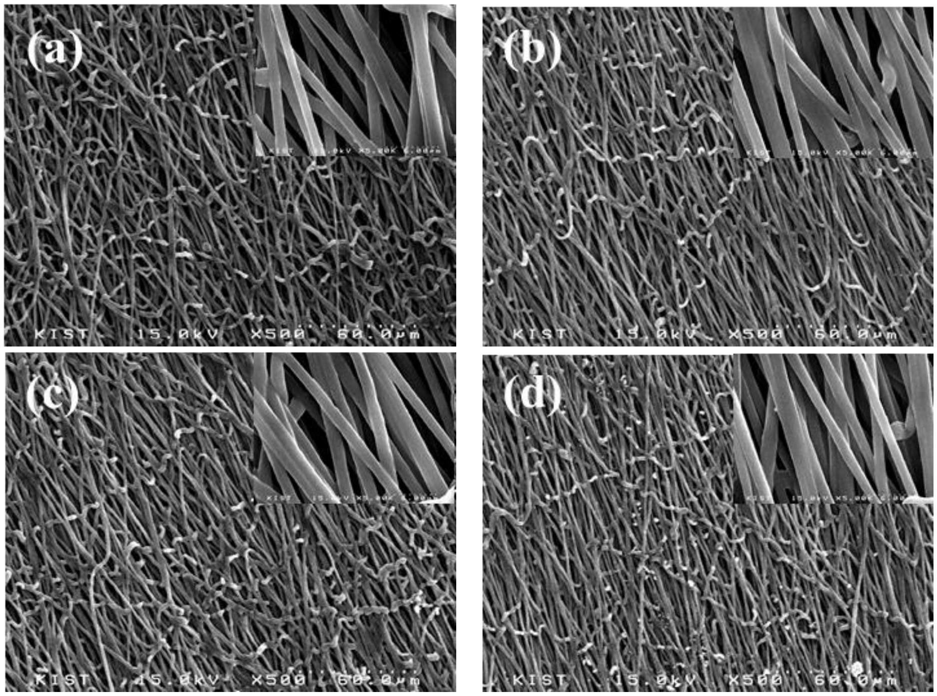

Figure S9. SEM images of the electrospun PEI/PVdF (1:1 wt. ratio) blend membranes (1/4 heat-pressed) after heat-stretching process followed; the stretching ratio (a) 60%, (b) 89% (c) 93% and (d) 100%
